# Considerations for biomarker-targeted intervention strategies for tuberculosis disease prevention

**DOI:** 10.1016/j.tube.2017.11.009

**Published:** 2018-03

**Authors:** Andrew Fiore-Gartland, Lindsay N. Carpp, Kogieleum Naidoo, Ethan Thompson, Daniel E. Zak, Steve Self, Gavin Churchyard, Gerhard Walzl, Adam Penn-Nicholson, Thomas J. Scriba, Mark Hatherill

**Affiliations:** a Vaccine and Infectious Disease Division, Fred Hutchinson Cancer Research Center, 1100 Fairview Ave N, Seattle, WA, 98109, USA; b Centre for the AIDS Programme of Research in South Africa (CAPRISA), Durban, Medical Research Council-CAPRISA HIV-TB Pathogenesis and Treatment Research Unit, Doris Duke Medical Research Institute, University of KwaZulu-Natal, Durban, South Africa; c Center for Infectious Disease Research, 307 Westlake Ave N #500, Seattle, WA, 98109, USA; d The Aurum Institute, Johannesburg, 29 Queens Road, Parktown, Johannesburg, Gauteng, 2193, South Africa; e School of Public Health, University of Witwatersrand, Johannesburg, South Africa; f Advancing Treatment and Care for Tuberculosis and HIV, South African Medical Research Council, Johannesburg, South Africa; g DST/NRF Centre of Excellence for Biomedical TB Research and SAMRC Centre for TB Research, Division of Molecular Biology and Human Genetics, Department of Biomedical Sciences, Faculty of Medicine and Health Sciences, Stellenbosch University, Cape Town, South Africa; h South African Tuberculosis Vaccine Initiative, Institute of Infectious Disease and Molecular Medicine, Division of Immunology, Department of Pathology, University of Cape Town, South Africa

**Keywords:** Correlate of risk, Biomarker, mRNA, Study design, Tuberculosis, 3HP, 12-dose once-weekly rifapentine 900 mg plus isoniazid 900 mg, ACS, Adolescent Cohort Study, COR, correlate of risk, CORTIS, Correlate of Risk Targeted Intervention Study, IGRA, interferon-gamma release assay, MTB, *Mycobacterium tuberculosis*, RR, relative risk, TB, tuberculosis, TST, tuberculin skin test

## Abstract

Current diagnostic tests for *Mycobacterium tuberculosis* (MTB) infection have low prognostic specificity for identifying individuals who will develop tuberculosis (TB) disease, making mass preventive therapy strategies targeting all MTB-infected individuals impractical in high-burden TB countries. Here we discuss general considerations for a risk-targeted test-and-treat strategy based on a highly specific transcriptomic biomarker that can identify individuals who are most likely to progress to active TB disease as well as individuals with TB disease who have not yet presented for medical care. Such risk-targeted strategies may offer a rapid, ethical and cost-effective path towards decreasing the burden of TB disease and interrupting transmission and would also be critical to achieving TB elimination in countries nearing elimination. We also discuss design considerations for a Correlate of Risk Targeted Intervention Study (CORTIS), which could provide proof-of-concept for the strategy. One such study in South Africa is currently enrolling 1500 high-risk and 1700 low-risk individuals, as defined by biomarker status, and is randomizing high-risk participants to TB preventive therapy or standard of care treatment. All participants are monitored for progression to active TB with primary objectives to assess efficacy of the treatment and performance of the biomarker.

## 1 Introduction

Approximately 1.7 billion individuals worldwide are infected with *Mycobacterium tuberculosis* (MTB) [1]. These individuals are identified by a positive tuberculin skin test (TST) or interferon-gamma release assay (IGRA) that indicates an MTB-specific immunological response. Though IGRA/TST-positive individuals have higher risk of developing TB disease than uninfected individuals [2], the tests have poor specificity for identifying individuals who will progress to active TB, since over 95% of IGRA/TST-positive HIV-uninfected and approximately 70% of IGRA/TST-positive HIV-infected individuals never develop active disease [3], [4]. Therefore, although prevention of progression from infection to TB disease is key to achieving WHO elimination targets [5], mass preventive therapy based on IGRA/TST screening in TB endemic countries would need to treat 50–80% of the population, most of them unnecessarily. Using current commercial IGRAs, such as the QuantiFERON-TB Gold In-Tube test, it has been estimated that 85 persons with latent TB would need to be treated to prevent a single case of active TB [6], making such an approach neither viable nor cost-effective. Mass preventive therapy for all MTB-infected people would also not be effective, since reinfection could occur before programmatic coverage could be completed. For instance, the Thibela trial enrolled South African mine workers, 89% of whom were estimated to have latent MTB infection [7]; the mass test-and-treat strategy in this population had no significant effect on TB incidence [8].

A screening tool with greater specificity could identify individuals with the highest risk of developing TB disease, thereby reducing the number of people who would be treated needlessly in an IGRA-targeted treatment campaign. A risk-targeted test-and-treat strategy based on a highly specific biomarker would also impact the greater epidemic by identifying and treating persons with infectious TB disease, thereby interrupting transmission.

**Fig. 1:**
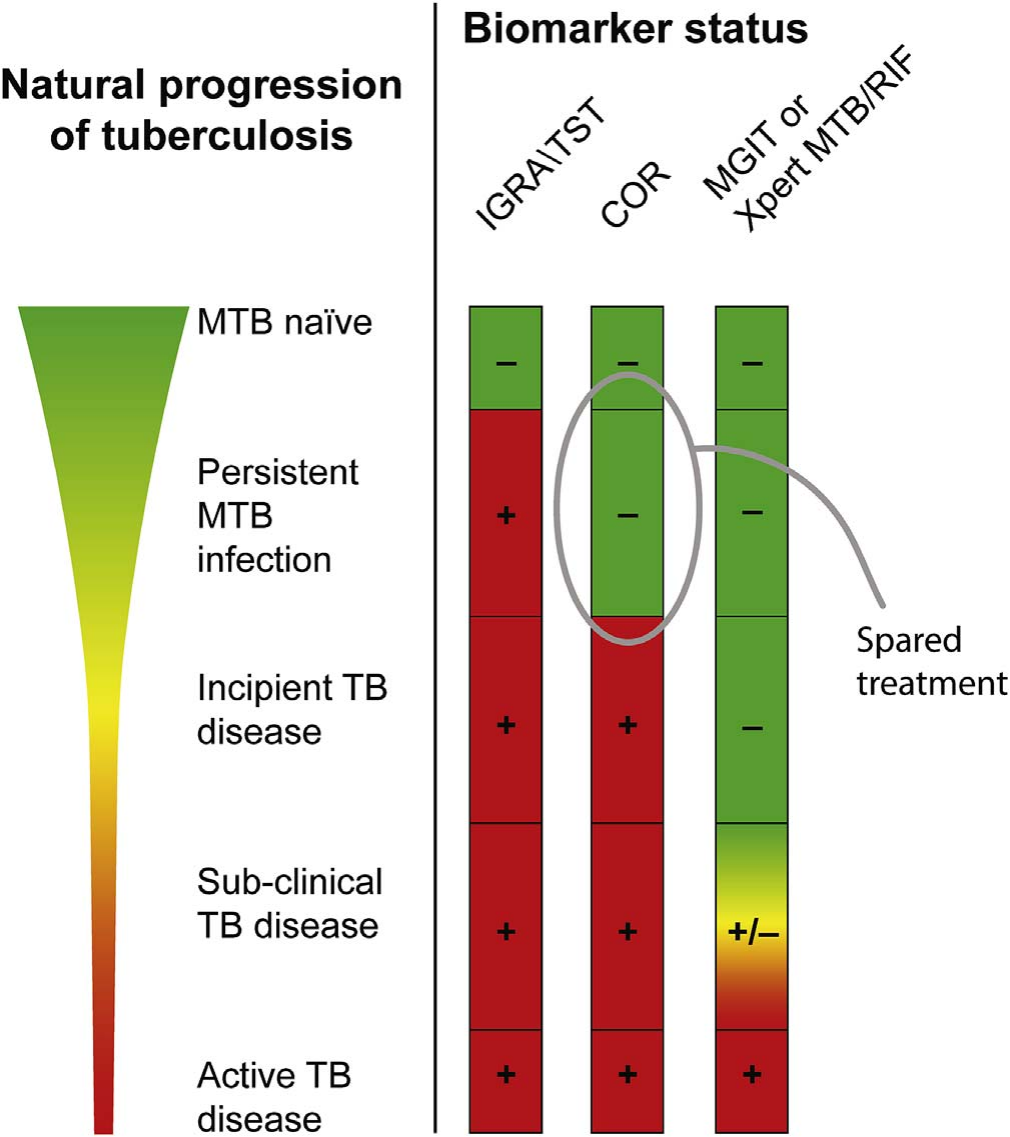
**Tuberculosis biomarker comparison.** Infection with *Mycobacterium tuberculosis* (MTB) is associated with a broad clinical spectrum of pathogenesis that includes asymptomatic persistent MTB infection; incipient TB disease; asymptomatic, but microbiologically detectable, sub-clinical TB disease; and active symptomatic TB disease. In CORTIS-01 the TB disease endpoint is based on detection of MTB nucleic acid (Xpert MTB/RIF) and/or sputum culture (Mycobacteria Growth Indicator Tube assays, MGIT), a standard tool for TB diagnosis. The interferon gamma release assay (IGRA) and the tuberculin skin test (TST) detect MTB-specific T cells in the blood and are currently used to identify MTB-infected individuals. Most IGRA/TST+ individuals will not develop active disease. The COR is an mRNA expression signature that detects the type I/II interferon response and is associated with active disease (i.e. diagnostic) and with individuals who are likely to develop disease (i.e. prognostic). A test-and-treat strategy would treat only COR+ individuals to prevent active disease. Individuals with latent MTB infection that have a lower risk of developing active TB disease may be IGRA/TST+ and COR-, thus sparing them unnecessary treatment in a mass test-and-treat campaign.

MTB infection is associated with a broad clinical spectrum of disease pathogenesis [9], including an asymptomatic stable quiescent infection state; an incipient pre-clinical disease state; an asymptomatic, but microbiologically detectable, subclinical disease; and an active symptomatic disease state [6] (Fig. 1). For biomarker evaluation we define TB disease and TB risk by that which is microbiologically detectable, a standard for TB diagnosis. One consequence is that many individuals with TB disease may not have presented for medical care (i.e. the walking ill), creating the need for two types of cases in the setting of a clinical trial with active surveillance: (1) Prevalent cases detected at the beginning of a study are either subclinical or active, but previously undiagnosed; and (2) Incident cases, which progress to active disease during follow-up. A biomarker's ability to identify individuals with prevalent TB (i.e. diagnostic performance) and its ability to predict which individuals will develop TB (i.e. prognostic performance) may differ, though both are clinically valuable for TB prevention.

Previously we identified and validated a prognostic blood mRNA expression signature that could be used in a risk-targeted TB prevention campaign [10] (correlate of risk, COR). In a prospective cohort of HIV-uninfected South African adolescents, the COR had 84% specificity (95% CI 79.1–87.9) and 71% sensitivity (95% CI 55.9–83.1) in combined training and test sets for discriminating between TB progressors and non-progressors within 12 months of testing [11]. Sensitivity was greater nearer to the time of TB diagnosis. The COR also displayed comparable or better performance for discriminating active TB disease from latent MTB infection in published microarray data from adult cohorts in the UK, South Africa, and Malawi [10], and in our own preliminary data (92% sensitivity and 93% specificity for prevalent TB disease) [11]. These findings demonstrate that the COR is robust to age and geographic region in its potential to detect prevalent TB, in addition to its ability to identify persons at risk for developing active (incident) TB disease.

The use of a host biomarker to target high-risk individuals for TB preventive therapy is a form of personalized medicine similar to blood-based biomarkers that guide treatment selection decisions in cancer therapy [12], [13], [14], [15]. Risk-targeted treatment is a potential TB control strategy that can be clinically evaluated with existing treatment selection paradigms that can be adapted to address the unique challenges of TB epidemiology, pathophysiology and available interventions [16], [17]. For successful risk-targeted treatment, it is important to have both 1) an efficacious preventative treatment and 2) a good biomarker for selecting high-risk individuals. In treatment selection trial designs, both factors are evaluated concurrently.

A successful mass campaign to halt TB transmission using a risk-targeted test-and-treat strategy would require an effective and safe short-course, sterilizing preventive therapy, such as the 12-dose once-weekly rifapentine 900 mg plus isoniazid 900 mg (3HP) regimen [18], [19]. This regimen has been shown to be as effective as 9 months of isoniazid alone at preventing TB disease in latently infected individuals [19]; however, more data are needed to determine its efficacy in a biomarker-selected high-risk subgroup and in hyper-endemic settings with a high force of infection. Before a large cluster randomized trial is implemented to evaluate population level effects of the strategy, a proof-of-concept trial is needed to: (1) validate the COR in an unselected population, (2) test the efficacy of treating COR^+^ persons in reducing incident TB disease, and (3) evaluate the overall value of a risk-targeted test-and-treat strategy with these operating characteristics.

Here we discuss general considerations for a proof-of-concept study and the specific design of the Correlate of Risk Targeted Intervention Study (CORTIS-01; ClinicalTrials.gov NCT02735590), a clinical trial currently underway in TB hyper-endemic areas of South Africa. CORTIS-01 enrols COR+ and COR-individuals and randomizes COR + individuals to TB prevention therapy (3HP) or standard of care (SOC). The objective of this manuscript is to provide justification of the CORTIS-01 trial design and to inform future efforts to design trials of targeted TB interventions. These considerations become increasingly important as the results of CORTIS-01 become available and other biomarkers for TB are developed. Applied at the community level, such risk-targeted strategies are anticipated to accelerate reduction in TB incidence and mortality and to reduce the pool of MTB-infected individuals independently of ongoing improvements in TB vaccines, diagnostics, and therapeutics.

## 2 General design considerations for evaluating screen and treat strategies

Several clinical trial designs have emerged for evaluating the benefits of biomarkers employed in treatment selection strategies in cancer and personalized medicine [16]. Under the gold standard trial design all participants are randomized to treatment or no treatment, regardless of biomarker status. In the context of TB prevention, such a design would enroll four groups of participants, where COR ^±^ denotes TB risk status based on a dichotomous COR biomarker: (1) Treated, COR^−^, (2) Untreated, COR^−^, (3) Treated, COR^+^, and (4) Untreated, COR^+^ (Fig. S2). The scientific advantage of this design is that treatment efficacy can be directly compared in high and low risk populations, and *post hoc* analyses can be used to optimize a biomarker threshold or to compare different biomarkers head-to-head (e.g. an IGRA), since all participants are subject to randomization. The specific proportions of COR^+^ and COR^−^ participants enrolled in a given trial have important logistical and ethical implications for the conduct of the trial. For example, enrolling and randomizing all members of a community to treatment is both logistically challenging and ethically problematic since it would require following and treating large numbers of low-risk COR^−^ participants, who are unlikely to receive any benefit from treatment, while being subject to its risks. To address this limitation, three alternative clinical trial designs have emerged in the cancer field for evaluation of biomarker-guided therapy [20].

An “enrichment design” would enrol only high-risk individuals that are COR^+^ and randomize participants to treatment or no treatment. This design enables evaluation of treatment efficacy in a high-risk subgroup and benefits from a much smaller sample size, however it precludes evaluation of biomarker performance, since COR^−^ participants are not enrolled.

In a “biomarker strategy” design, the test-and-treat *strategy* is randomized to study participants; participants in one group are provided the standard of care (SOC, i.e. no risk-targeted treatment strategy) while participants in the other are provided treatment based on their biomarker status (i.e. treatment for COR^+^ only). This design enables direct evaluation of the efficacy of the test and treat strategy in comparison to the SOC. In the context of TB it could also be implemented as a community-based cluster randomization of the strategy to capture indirect effects like transmission reduction or knowledge of COR status. Such cluster randomized trials would also potentially be more feasible in the real-world setting. However, like the fully randomized trial, a large fraction of enrolled participants contribute very little information to estimates of treatment efficacy, which refers to the relative reduction in risk in treated compared to untreated COR^+^ participants; COR^−^ participants enrolled in both the SOC and intervention arm may even be redundant.

Finally, a “hybrid” design is an enrichment design that additionally includes a limited biomarker-negative group to gain information about biomarker performance. This design affords randomized assessment of treatment efficacy, efficient estimation of biomarker performance, and preliminary estimates of strategy efficacy. The design does not permit the comparison of test-and-treat strategies using different biomarkers (e.g. IGRA vs. COR), since a given participant can only be recommended for treatment using one biomarker. However, a direct comparison can be made of the biomarkers' abilities to discriminate TB progressors from non-progressors. The most appropriate design would also depend on the prevalence and performance of the biomarker in the target population and the strength of the evidence indicating that only the biomarker-positive subgroup will benefit from the treatment [21], [22]. The challenge of TB control in high transmission settings is the high prevalence of latent MTB infection and the low proportion that progress to TB disease, which calls for innovative and expeditious designs. Considering these factors, CORTIS-01 was based on a hybrid design.

## 3 Ethical considerations

The basic underpinning of the ethical considerations for any clinical trial is that the potential benefits outweigh the potential risks for each participant and thus a hybrid design in which COR^+^ individuals predicted to be at higher risk of TB disease are not allocated treatment is potentially contentious. In countries such as South Africa where there is a high TB burden and the majority of adults have latent MTB infection (defined by a positive IGRA or TST), the protective benefits of therapy for latent MTB infection are transient, and limited to the fraction of recipients that will progress to TB disease during their lifetime. The risks of therapy are substantial and potentially include treatment intolerance, hepatoxicity and a high pill burden. Accordingly, treatment of latent MTB infection is not the SOC for TST or IGRA positive individuals in South Africa, except for children under the age of five, HIV-infected persons and people with silicosis [23]. We hypothesize that the COR has better prognostic specificity for TB disease than IGRA/TST, but like IGRA/TST, most COR^+^ individuals will remain disease-free and would not benefit from preventative therapy. Individuals with “false positive” COR^+^ status would thus only be exposed to the potential risk of preventative therapy, not its benefits. Therefore, it is important to consider the body of knowledge about a biomarker and the precision of its performance in the study population. Estimates of COR prognostic performance are based on two nested case-control studies (n = 46 progressors and 107 matched controls from the Adolescent Cohort Study (ACS) [24] and n = 73 progressors and 301 controls from the GC6 household contact study [10]); COR performance has never been validated in a prospective cohort. Thus, individual risk of COR^+^ status is not yet known. Moreover, COR performance may differ in cohorts differing in age or geography. There is also uncertainty in how COR^+^ individuals will respond to preventative therapy, since its benefit has not been demonstrated in this biomarker-defined subgroup. Since the COR seems to identify participants with incipient TB disease, it is possible that the regimen will not be as effective at preventing disease progression as it is in clearing a latent infection, which may reduce treatment efficacy in COR^+^ individuals. COR^+^ individuals may also be more likely to have risk factors for TB disease such as a weakened immune response, which may also reduce treatment efficacy. It is possible that COR^+^ individuals require a full course of 4-drug curative TB treatment rather than 3HP to prevent progression to disease, but evidence of (1) good prognostic COR performance, and (2) poor efficacy of 3HP preventative therapy would be needed to justify such a major intervention for otherwise healthy people. With knowledge of biomarker performance and treatment efficacy, the number of biomarker-targeted treatments needed to avert one case is critical in finding the balance of potential risks and benefits to participants. Based on the cumulative knowledge and uncertainty summarized above we believe there is equipoise for the CORTIS-01 design and we are hopeful that the results will provide more knowledge for the design of future studies and interventions.

It is possible that the biomarker will perform so well that it is ethically imperative to provide immediate treatment to all COR^+^ participants before the end of follow up. If the study treatment is demonstrating sufficient efficacy then it could be provided to all COR+ participants, otherwise a more rigorous therapeutic regimen could be recommended. Planning an unblinded interim analysis of treatment efficacy and biomarker performance protects participant welfare in these scenarios. Such an analysis can also be used to halt the trial for futility if low rates of TB or low biomarker performance precludes the possibility of meeting any of the study objectives. Ideally the analysis would assess appropriate stopping thresholds for biomarker performance and treatment efficacy and would be triggered by a pre-specified total number of TB cases that would provide sufficient power to detect these outlier scenarios (see Supplement).

## 4 CORTIS considerations

### 4.1 CORTIS objectives

**Fig. 2:**
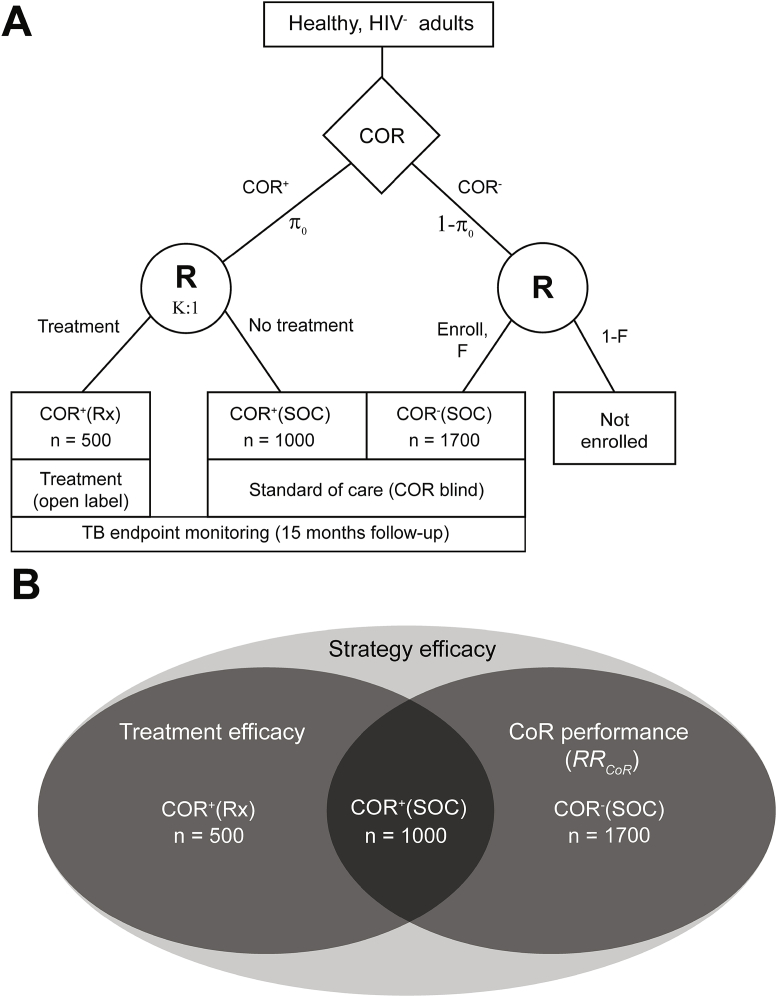
**“Hybrid” treatment selection design for CORTIS-01.** (A) Healthy, HIV-uninfected adults are recruited and screened using the COR blood-based biomarker. Within 28 days of screening, COR^+^ participants are enrolled and randomized to preventive therapy (Treatment/Rx) or standard of care (SOC). A fraction (*F*) of COR^−^ participants are enrolled in a SOC arm. All enrolled participants are followed for 15 months for TB disease. COR^+^ and COR^−^ participants are enrolled concurrently and therefore rate of enrolment directly depends on the prevalence of COR-positivity (*π*_*0*_). (B) The rate of incident TB in the COR^+^(Rx) and COR^+^(SOC) groups can be compared to evaluate treatment efficacy, while comparison of the COR^+^(SOC) and COR^−^(SOC) groups yields information about biomarker performance. Strategy efficacy is assessed using all three groups.

The three fundamental objectives of a CORTIS are to: (1) Evaluate the diagnostic performance of the COR for identifying prevalent TB disease and the prognostic performance of the COR for predicting incident TB disease, (2) Evaluate the efficacy of the treatment regimen (in CORTIS-01, the short-course 3HP treatment regimen) to reduce incident TB in COR^+^ participants, and (3) Evaluate the efficacy of the test-and-treat strategy in the study population as a whole. Each objective relies on the comparison of TB risk in two comparator groups (Fig. 2).

Biomarker performance can be evaluated by comparing the relative risk of TB disease among COR^+^ versus COR^−^ participants (RR_COR_) while receiving the SOC (i.e. active surveillance for symptomatic TB); this can be computed based on prevalent or incident TB for evaluating diagnostic or prognostic performance, respectively. Treatment efficacy is the relative reduction in TB incidence in treated COR^+^ compared to untreated (i.e. receiving the SOC) COR^+^ participants; treatment efficacy may be different in COR^+^ individuals compared to IGRA^+^ individuals for the reasons discussed above. The efficacy of the test-and-treat strategy is determined by estimating the relative reduction in TB incidence in the entire study population had all COR^+^ participants been provided the treatment compared to a study population that had not received any risk-targeted treatment. To make these comparisons for all three objectives, at least three groups of participants need to be enrolled, evaluated for prevalent TB at baseline, and followed by active surveillance for incident TB disease: COR^+^(Rx), randomized to treatment; COR^+^(SOC), randomized to SOC only; and COR^−^(SOC), provided SOC only.

A hybrid trial designed with only these groups has certain limitations. For instance, without including a placebo for evaluation of treatment efficacy, estimates could be biased by knowledge of treatment assignment or knowledge of COR status, if only one of the groups has such knowledge. For example, knowledge of COR status or treatment could influence a participant's behavior and also their TB risk, but including such behavior modifications in the estimation of the treatment effect is permissible because it would also presumably affect a real-world strategy implementation. Similarly, for estimation of RR_COR_, knowledge of COR status by either COR^−^ or COR^+^ participants could introduce bias. For CORTIS-01 we chose not to use a placebo, but instead enrolled an untreated COR^+^ group assigned to SOC active surveillance, and double-blinded to COR status [this is achieved operationally by blinding all site staff and participants to COR status with the exception of participants in the COR^+^(Rx) group]; the group can be shared for estimation of treatment efficacy and RR_COR_. While there is potential for knowledge of open-label treatment and COR^+^ status in the COR^+^(Rx) group to affect treatment efficacy and strategy efficacy estimation, it is permissible for these effects to be included, as they would also contribute to overall effectiveness. We have also considered that knowledge of COR^+^ and open-label treatment status might introduce investigator ascertainment bias of TB cases. In order to minimize this potential bias, we ensure that different site staff are responsible for dispensing therapy and collecting TB symptom data, which would trigger an investigation for TB endpoint determination. Moreover, TB screening at study visits is the same in all arms to avoid differences in ascertainment of TB cases. The advantage of a three-arm trial is a substantial increase in statistical power per participant, since participants and TB endpoints are effectively shared across the three objectives. This highly efficient trial design allows substantial savings in study resources, duration, and cost, since fewer total participants are required. Group sizes can be further optimized, based on hybrid design simulations described below, to reduce the fraction of COR^−^ individuals enrolled.

### 4.2 TB endpoint assessment

The CORTIS-01 TB disease endpoint is based on the detection of MTB nucleic acid (Xpert MTB/RIF) and/or culture (Mycobacteria Growth Indicator Tube assays), requiring two positive results of either test from two serially-collected sputum samples. At enrolment, two sputum samples are collected from all participants able to produce sputum for Xpert MTB/RIF and/or culture; thereafter presence of compatible symptoms triggers investigation for TB during follow-up. Finally, all sputum-productive participants are tested again at end of study to detect any cases of sub-clinical disease (Fig. 1). We defined prevalent TB cases as those detected at the first (enrolment) study visit with all others defined as incident TB. We separately evaluate the COR as a prognostic biomarker of progression to incident disease and as a diagnostic biomarker of prevalent TB disease. In the future, a biomarker with both prognostic and diagnostic utility might even leverage two different triage test thresholds, one to trigger investigation for active disease and another for provision of treatment of infection (Fig. S1A). This additional capability would be a substantial benefit to a test-and-treat program.

The evaluation of COR performance and strategy efficacy could also include both prevalent and incident TB cases, since both are examples of undetected TB in the community that could benefit from detection and treatment. Evaluation of treatment efficacy will be conducted only on incident TB (i.e. a modified intent-to-treat analysis), as TB cases diagnosed at baseline will be withdrawn from the study and referred for curative therapy.

### 4.3 Biomarker operationalization

In preparation for CORTIS, we established high-throughput, automated standard operating procedures for the PAXgene whole blood RNA extraction and refined microfluidic multiplex qRT-PCR (Fluidigm BioMark HD platform) steps to simultaneously measure the COR signature in 94 samples, plus a no-template control and a standardized internal positive control (IPC) sample. The locked down analysis script includes quality control (QC) filters for exclusion of individual samples, single transcripts or entire qRT-PCR runs, should they be flagged deviating from pre-defined criteria. Robustness of COR performance was assessed, including evaluation of assay accuracy and precision and measurement of inter -assay, intra-assay and inter-operator variance. The COR assay had excellent repeatability and reproducibility, while also incorporating a standardized IPC sample into each qRT-PCR run, which must meet pre-specified performance targets (see Supplement). The COR assay displays robust performance and reliable interpretation even at scale, enabling centralized laboratory testing of >400 samples per week.

### 4.4 Biomarker performance estimation for study simulations

For the design of CORTIS-01 it was important to consider that the ability of the COR to predict TB progression was higher when the COR was measured more proximally to TB diagnosis. In other words, the CORs ability to predict incident TB decreases over time, with a substantial decrease in performance after 18 months have elapsed [10]. We hypothesize that this decay in COR performance over time is related to the strength of the interferon response, which is more pronounced at sampling times proximal to active TB disease [25]. In the CORTIS we expect that a COR measured at baseline will be more effective at predicting incident TB earlier in the follow-up period; TB progression later in the follow-up period may be equally likely in participants who were COR^+^ or COR^−^ at baseline. Therefore, cumulative estimates of COR performance and strategy efficacy may decline with longer follow-up durations. However, the duration of follow-up must be sufficient to estimate the durability of treatment efficacy and to demonstrate the ability of the COR to predict development of active TB in the future. These factors directly impact the durability of the overall strategy, which will be an important criterion for an efficient and effective mass test-and-treat campaign.

**Fig. 3:**
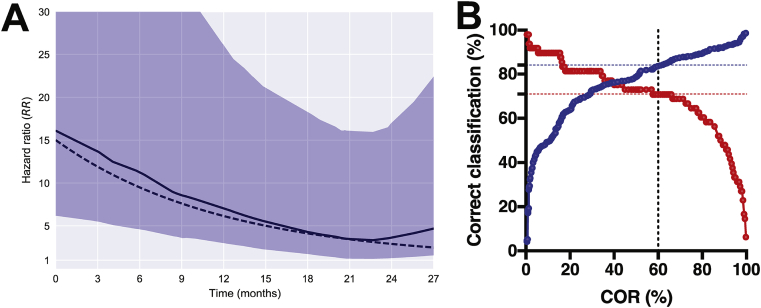
**Performance characteristics of COR biomarker.** The COR was measured at baseline in participants of the Adolescent Cohort Study (ACS). (A) Relative risk of developing TB disease for COR^+^ vs. COR^−^ participants was computed longitudinally using a cumulative incidence-based approach (blue shaded 95% confidence interval from bootstrap sampling). Data was approximated well by exponential decay from RR = 15 to RR = 1 with decay time constant of 12 months (dashed line). (B) Sensitivity (red) and specificity (blue) of COR in the ACS training and test set samples up to 1 year preceding TB disease. Classification performance depends on the threshold applied to the continuous readout. Using a 60% threshold, the COR had 71% sensitivity and 84% specificity.

To precisely estimate COR performance over time we re-analyzed data collected in the ACS [24] (see Supplement). We estimated the hazard ratio (HR) curve over time: the relative risk of COR^+^ versus COR^−^ participants (Fig. 3A). The shape of the HR curve was consistent with previous findings: at baseline, COR^+^ participants were 15-fold more likely to progress to active TB, and their relative risk for progression declined over time. The apparent increase in HR after 24 months is an artefact, due to a sharp decrease in the number of at-risk participants near study end. The HR curve was approximated well by an exponential decay model with a decay time-constant of 12 months. Since the original study included only IGRA^+^ individuals, we also measured the COR in 100 IGRA^−^ individuals; reported results reflect a re-weighting for translation to a mixed IGRA ^±^ population.

Multiple HR curves were estimated using a range of biomarker thresholds for determining COR positivity. An optimal threshold of 60% was selected for the trial, due to the relatively high sensitivity/specificity (Fig. 3B) and the estimated 15% population prevalence of COR^+^ individuals (Fig. S1B), which would be a reasonable target population for implementation of a test-and-treat program. We note that the choice of a relatively low COR threshold also provides more flexibility for *post hoc* analyses in the CORTIS trial that evaluate the impact of higher test thresholds on diagnostic and prognostic performance. A higher COR threshold does not allow for *post hoc* analyses of a hypothetical lower threshold, since participants with a COR below the actual threshold would not have been randomized to the COR^+^ groups.

### 4.5 CORTIS objective evaluation

Each CORTIS objective requires a specific hypothesis and accompanying statistical test. Studies employing a time-to-event endpoint, such as TB progression, commonly hypothesize that the endpoint rate is different in two comparator groups and test this using a Cox proportional hazards regression [26]. For example, one could evaluate COR performance by comparing the rate of incident TB in COR^+^ versus COR^−^ groups (both untreated) and testing for a significant difference with a Cox model. Interpretation of the coefficients derived from a Cox regression and their significance requires the assumption that TB incidence is proportional in the two groups, yet relative risk of COR^+^ and COR^−^ participants' changes over time (Fig. 3A), thus falsifying this assumption. Therefore, we elected to use cumulative incidence-based statistics for the estimation of treatment efficacy, RR_COR_ and strategy efficacy, as they are not constrained by this assumption. These methods accommodate time-varying effects, allowing for visual assessment of the changes over time, as well as a direct test of the cumulative effect at the end of follow-up. Ultimately, it is the cumulative performance of the biomarker and the cumulative effect of treatment that are relevant for the implementation of a test-and-treat program; only a biomarker and treatment with long lasting effects will be clinically useful and meet the desired characteristics required for an effective mass campaign [6], [27]. Considerations for choosing follow-up duration include the need to assess treatment efficacy durability and the need to obtain precise estimates of the exponential decay in COR prognostic value.

Trials often test a null hypothesis of “no effect” as the lowest bar for establishing statistical significance. In CORTIS-01, we test more stringent hypotheses for treatment efficacy and COR performance to ensure that a statistically significant result is also clinically relevant. For COR performance, we defined a null hypothesis requiring that the cumulative relative risk of untreated COR^+^ versus COR^−^ participants over 15 months (RR_COR_(15)) is greater than 2 (two-sided, α = 0.05) (see Supplement). In addition to using RR(15), biomarker performance can be assessed using time-dependent estimates of sensitivity, specificity, positive predictive value, and number needed to treat [28]. Though cumulative effects may have the greatest impact on policy, monitoring these metrics longitudinally will offer important insights into the underlying pathogenesis and inform implementation of future risk-targeted prevention strategies. For treatment efficacy we require that cumulative efficacy over 15 months (TE(15)) is greater than 20%, however we use α = 0.1 (two-sided); relaxing the α-level is justified because previous studies have demonstrated the efficacy of the 3HP regimen for preventing TB disease and because less precision is required in this proof-of-concept study. A more definitive trial might require the more canonical α = 0.05.

To evaluate strategy efficacy, one would ideally compare cumulative incidence in two populations, each with a mixture of COR ^±^ status, only one of which is provided COR^+^ targeted therapy. Though these two groups do not exist explicitly, as a consequence of the “hybrid” design, they can be recreated *in silico* by sharing the COR^−^ group and scaling each COR^+^ group to the total COR prevalence (see Supplement). For strategy efficacy we use a more standard null hypothesis of no effect (α = 0.1, two-sided); the lower α-level is justified as the objective is to provide a preliminary estimate and demonstrate proof-of-concept for this novel prevention strategy.

### 4.6 Statistical power

To determine the number of participants that would be needed, we simulated trials using different group sizes and identified options with adequate power for the primary objectives (see Supplement for simulation details). Two operational parameters can be modified to alter the number of participants in each group: (1) the treatment randomization ratio (*K*) and (2) the fraction of screened COR^−^ participants to enrol (*F*). Though power can be increased overall by screening and enrolling more participants, a balance of power for all objectives is desirable. For example, we found that while randomizing more COR^+^ participants to treatment increased power for treatment efficacy, it reduced the total number of endpoints, tending to reduce power for RR_COR_. In future studies, if treatment efficacy has been well established, group sizes could be rebalanced to deemphasize the treatment efficacy objective. Decreasing the number of COR^−^ participants enrolled had little impact on power for RR_COR_ and strategy efficacy, until the number of projected endpoints was too low for precise estimates of incidence. Determining the duration of follow-up is complex, because as TB cases accrue over time, precision of treatment efficacy increases, but as RR_COR_ wanes, power for detecting significant cumulative RR_COR_ and strategy efficacy declines.

**Fig. 4:**
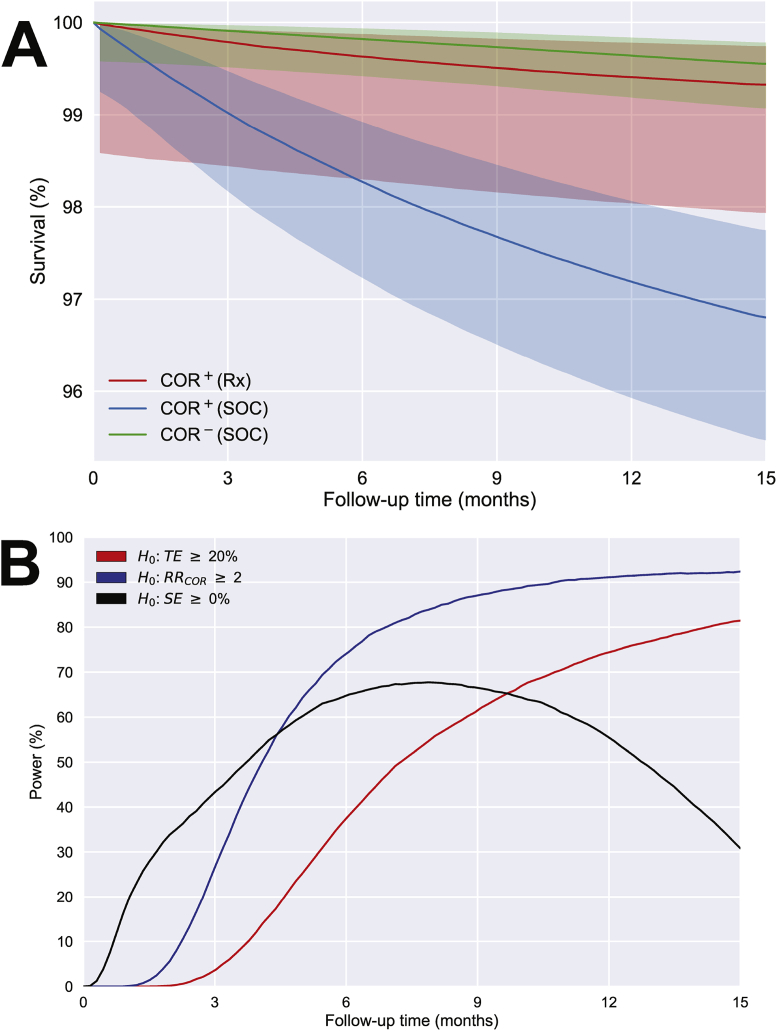
**Simulated TB endpoints.** CORTIS-01 was simulated stochastically 10 K times using parameters estimated from previous observation studies, including overall TB incidence of 1.1 cases per year, treatment efficacy of 80% and an initial COR relative-risk of 15, decreasing exponentially to 1 with decay constant of 12 months (see *Supplement*). (A) Kaplan-Meier survival curves and the associated 2.5th and 97.5th percentiles across simulations for the COR^+^(Rx) group (red), the COR^+^(SOC) group (blue) and the COR^−^(SOC) group (green) within each trial. (B) Power to detect statistically significant treatment efficacy (TE ≥ 20%; two-sided α = 0.1; blue line), relative-risk (RR_COR_ ≥ 2; two-sided α = 0.05; red line) and strategy efficacy (SE ≥ 0%; two-sided α = 0.1; black line) was computed as the fraction of simulated trials in which a significant effect was detected.

We determined that 1500 COR^+^ and 1700 COR^−^ participants should be enrolled and followed for 15 months and 500 COR^+^ participants should be provided 3HP preventive therapy (*K* = 0.5). Based on an estimated COR prevalence of 15% (Fig. S1B), this would require screening approximately 10,000 HIV-uninfected individuals, with 6800 COR^−^ individuals who would be eligible but not enrolled (*F* = 0.2). One operational challenge is that COR prevalence directly impacts the rate of enrolment and the total number of individuals who would need to be screened. Lower than expected COR prevalence may indicate greater biomarker specificity, however it creates a logistical challenge of having to screen more volunteers. Obtaining accurate knowledge of COR prevalence in the target population is critical to success. From the simulations we can estimate Kaplan-Meier “survival” curves for the TB endpoint in each group (Fig. 4A) and compute power for each objective. The study is designed to have 81% power for detecting significant treatment efficacy, 92% power for establishing a RR_COR_ > 2 and 30% power for detecting significant strategy efficacy (Fig. 4B; see Supplement). This balance reflects the relative importance of the objectives as well as the necessarily lower power for strategy efficacy, which was deemed an exploratory objective. In our simulations, power is relatively robust to reduction in follow-up, however the benefits of a lower cost and more expedient study need to be weighed against the loss of information about the durability of treatment efficacy and biomarker performance.

## 5 Implications and future directions

For future long-term country-wide strategies in which people undergo repeated (e.g. annual) COR testing to be realized, within-person COR variation will need to be characterized longitudinally. This is particularly relevant for COR^+^ participants who do not develop disease, as these represent false-positives that limit biomarker specificity. Longitudinal assessments could show that for these cases initial positivity was transient, possibly mediated by a non-TB induced interferon response (though we have shown that the COR differentiates TB disease from lung cancer, sarcoidosis, and pneumonia [10]). Longitudinal variation would also be relevant for understanding COR^−^ participants, as they would be tested regularly in a long-term strategy. Since COR performance at predicting active disease increases as the time of diagnosis approaches [10], it may be that repeated COR assessments have greater performance as latently infected individuals progress to active disease. A CORTIS with repeated longitudinal COR measurements could provide insight into these issues [29]. Future trials might also assess the performance of the COR to detect post-treatment recurrence, which ranges from 2 to 10% in hyperendemic communities [30], [31], [32], [33], or as a correlate of treatment response [10], [34], [35]. Future implementation studies could also be designed to estimate cost savings or adverse event reduction by comparing cost/adverse events of the biomarker-based test-and-treat strategy with those of alternative strategies. A final consideration is how HIV-infected individuals could be screened using the COR. In a pilot study, we determined that performance of the COR for discriminating prevalent TB disease from latent TB infection was 13% lower in HIV-infected individuals than in HIV-uninfected individuals (data not shown). We hypothesize that the reduced diagnostic performance may be attributed to the type I interferon signature observed in chronic HIV infection [36], [37]; it may thus be necessary to adjust the COR to account for HIV status to provide risk-targeted therapy to HIV-infected individuals.

The CORTIS design could be relevant to future trials aimed at other infectious diseases with relevant epidemiological and pathogenic features. For example, only about 20–30% of individuals infected with *Trypanosoma cruzi* develop active Chagas disease and current drugs for treating *T. cruzi* infection are highly toxic [38]. A CORTIS design could help identify and treat only individuals with the highest risk of progressing to disease, pending development of a drug regimen with demonstrated efficacy.

## 6 Conclusions

The CORTIS design addresses critical needs for the implementation of risk-targeted test-and-treat strategies by 1) evaluating the COR as a prognostic test for incident disease; 2) evaluating the efficacy of preventive therapy in COR^+^ individuals, and 3) providing a preliminary assessment of a test-and-treat strategy. The ethical and technical considerations discussed here may be relevant to the design of future trials of risk-targeted TB interventions as improved TB biomarkers are validated and may also provide a framework within which the CORTIS-01 results can be interpreted as they become available.

## Role of the funding source

The funders of CORTIS-01 had no role in designing CORTIS-01 or in the writing or submission of this manuscript.

## Declaration of interest

Conflicts of interest: AP-N, TJS, EGT and DEZ report a pending patent based on the gene signature, 4544P/GB: Biomarkers for prospective determination of risk for development of active tuberculosis. 11/08/2015.

## Appendix A Supplementary data

mmc1 mmc1
