# Supplementary material for: Considerations for biomarker-targeted intervention strategies for tuberculosis disease prevention
Source: Tuberculosis (Edinb). 2018 Mar;109:61–8. doi: 10.1016/j.tube.2017.11.009 (PMC5884308; doi:10.1016/j.tube.2017.11.009)
Supplement: mmc1 [file mmc1.pdf]

Supplementary Materials

Contents

Assay qualification 1
Biomarker performance estimation for study simulations 2
CORTIS objective evaluation 2
Treatment efficacy (TE) 2
COR performance 2
Strategy efficacy (SE) 3
Statistical power 3
Endpoint driven interim analysis 4
Supplementary Figures 5
Figure S1. Biomarker properties. 5
Figure S2. Clinical trial designs. 6
Figure S3. Evaluation of CORTIS objectives in simulated trials. 8
Supplementary Table 9
Table S1. Simulation Parameters 9
Supplementary References 9

Assay qualification

We incorporated quality control (QC) criteria into the computational script used for analysis of the qRT-PCR data, based on the distribution of cycle threshold (Ct) values obtained for each primer/probe set, detection of reference (housekeeper) transcripts and number of transcripts detected. This QC filter enables exclusion of data for individual transcripts or for an entire sample if QC criteria are not met. We also evaluated the range and linearity of the COR assay, and determined that a minimum of 25 ng input RNA is required. By measuring median concentrations or RNA from over 200 donor PAXgene tubes, we established that a standardized volume of eluted RNA could be used for the PCR test, alleviating the need to perform repetitive RNA quantitative/qualitative steps. We also performed extensive accuracy and precision experiments to measure inter-assay, intra-assay and inter-operator

variance. Briefly, three operators used a standard volume of RNA to perform cDNA synthesis, pre-amplification and multiplex qRT-PCR on 6 replicates of each of 8 donor samples, on 4 separate 96.96 Fluidigm chips run on different days (24 tests per donor for each operator). A range of donors representing samples scoring COR^{high}, COR^{med} and COR^{low} was selected. Correlation testing of repeatability and reproducibility was excellent. Specifically, all Spearman's correlation rho values comparing each condition with the others were >0.9, and all p values were < $1x10^{-8}$ (data not shown). Finally, we assessed assay robustness by deliberately introducing small variations in method parameters. The COR test was remarkably robust and these variations did not change the COR score markedly. A manuscript detailing the assay qualification results is currently in preparation.

Biomarker performance estimation for study simulations

To precisely characterize COR performance over time we reanalyzed data from the Adolescent Cohort Study [1]. We focused our analysis on biomarker levels measured in baseline samples and evaluated their relationship with incident TB as a time-to-event endpoint, using standard survival analysis methodology. Briefly, the ACS collected data on TB cases (N = 47) and covariate matched controls in a 2:1 ratio using sex, school, age and ethnicity (N = 105). To recover HR estimates for the entire population we used inverse probability weighting (IPW) of the covariate-defined strata in a two phase survival analysis. A 95% confidence interval was estimated using bootstrap samples of the phase 2 data. For reweighting of the results for translation to a mixed $IGRA^{+/-}$ population, an estimated 14% IGRA prevalence was assumed. The conservative assumption was also made that the biomarker is completely ineffective (RR_{COR} = 1) in $IGRA^{-}$ individuals.

CORTIS objective evaluation

Treatment efficacy (TE)

In CORTIS-01, the following null hypothesis is tested to evaluate treatment efficacy:

$$H_0: TE(15) \leq 20\% \text{ (two-sided, } \alpha = 0.1)$$

where H_0 is the null hypothesis and HX is the cumulative incidence estimated for the specified group using the Product-Limit estimator of Nelson-Aalen [2]. A confidence interval is computed for TE based on Greenwood's formula [3, 4].

COR performance

In CORTIS-01, the following null hypothesis is tested to evaluate COR performance:

$$H_0: RR_{COR}(15) < 2 \text{ (two-sided, } \alpha = 0.05)$$

$$RR_{COR}(15) = H_B/H_C$$

Strategy efficacy (SE)

Similar to TE, SE(15) is one minus the ratio of the cumulative incidence in these two populations:

$$SE(15) = 1 - H_{RTT} / H_{SOC}$$

where H_{RTT} is cumulative incidence in the risk-targeted therapy population and H_{SOC} is that under the standard of care, which can be expressed as cumulative incidences in COR^+ and COR^- weighted by COR prevalence at baseline (π_0):

$$H_{RTT} = \pi_0 (H_{RTT+}) + (1 - \pi_0)(H_{RTT-})$$
$$H_{SOC} = \pi_0 (H_{SOC+}) + (1 - \pi_0)(H_{SOC-})$$

Note that H_{RTT+} is the cumulative incidence of COR^+ participants who were provided preventive therapy. SE is a quantity that follows naturally from the fully randomized design described above requiring randomization of the strategy and enrolment of four independent groups. In the more efficient hybrid designed CORTIS, we assume that H_{RTT-} and H_{SOC-} are approximately equal and we substitute $H_{COR+(Rx)}$ for H_{RTT+}, $H_{COR+(SOC)}$ for H_{SOC+} and $H_{COR-(SOC)}$ for H_{SOC-} then SE can be expressed as:

$$SE(15) = 1 - [(1 - TE(15)) + \theta] / (1 + \theta)$$

where:

$$\theta = [(1- \pi_0) / \pi_0] / RR_{COR}(15)$$

This expression illustrates that SE is a combined evaluation of the biomarker and the preventive therapy to estimate the overall effect of a risk-targeted treatment strategy. We used the delta method to derive confidence intervals and a p-value. Since SE is impacted by imprecise estimates of TE and RR_{COR} and there is no prior evidence of an efficacious risk-targeted study, we use a null hypothesis of no effect:

$$H_0: SE(15) = 0 \text{ (two-sided, } \alpha = 0.1)$$

Statistical power

We used stochastic simulations of the trial to evaluate various designs and parameter values (**Figure S3**). The rates of TB progression in the simulation were based on the overall rate among South African adolescents and the prevalence and performance of the COR as measured in [5]. Though treatment efficacy may be >90%, it was conservatively assumed to be 80% for powering the study. The remaining simulation parameters are provided in **Table S1**; scripts for running the simulations are available upon request. Estimates of TE, RR_{COR} and SE were computed with data from each simulated trial and the statistical power for each objective was based on 10K repeated simulations.

From the simulations we found that higher values of *K* (more COR^+ participants randomized to treatment) resulted in more precise estimates of TE, however there were also fewer TB cases, which counteracted this effect and reduced power for the other objectives. Increasing *F* helped make trials more efficient as COR^- participants generally contributed little power to the RR_{COR} and SE objectives. Holding the total number of participants constant, increasing *F* allows more COR^+ participants to be enrolled, however power for RR_{COR} and SE declines as the number of cases in the COR^- group is too low to provide a precise estimate of incidence in that group. Determining the duration of follow-up is complex; as cases accrue, precision of TE increases, but as RR_{COR} wanes, power for detecting significant cumulative RR_{COR} and SE declines.

Endpoint driven interim analysis

For CORTIS-01 we determined that with 40 incident TB cases there would be sufficient power to detect two extreme treatment performance scenarios (highly efficacious treatment and highly inefficacious treatment), making this number a suitable trigger for the interim analysis. For the scenario in which the treatment is highly efficacious and all COR^+ participants could be provided treatment, we will jointly test that $RR_{COR}(X) > 5$ and $TE(X) > 60\%$. For the scenario in which treatment is not efficacious and all COR^+ participants should be referred to an alternative, more rigorous treatment, we will jointly test that $RR_{COR}(X) > 5$ and $TE(X) < 60\%$. Power for both scenarios is quite low; this is acceptable because the main objective is to complete the trial except under very high certainty of either of the two scenarios.

Supplementary Figures

Figure S1. Biomarker properties. (A) COR prevalence in an unselected population. The proportion of randomly selected $IGRA^-$ (blue) and $IGRA^+$ (red) healthy 17 and 18 year olds in the Worcester region of the Western Cape, who are classified as COR^+ at a range of score thresholds for COR. (B) COR model % positive votes in individuals with prevalent TB; incident TB within 1 year after sampling; incident TB in the 2nd year after sampling; and healthy controls (unpublished data pooled from several studies). The 80% vote threshold appears optimal to target investigation for active TB disease; and the 60% vote threshold appears optimal to target preventive therapy.

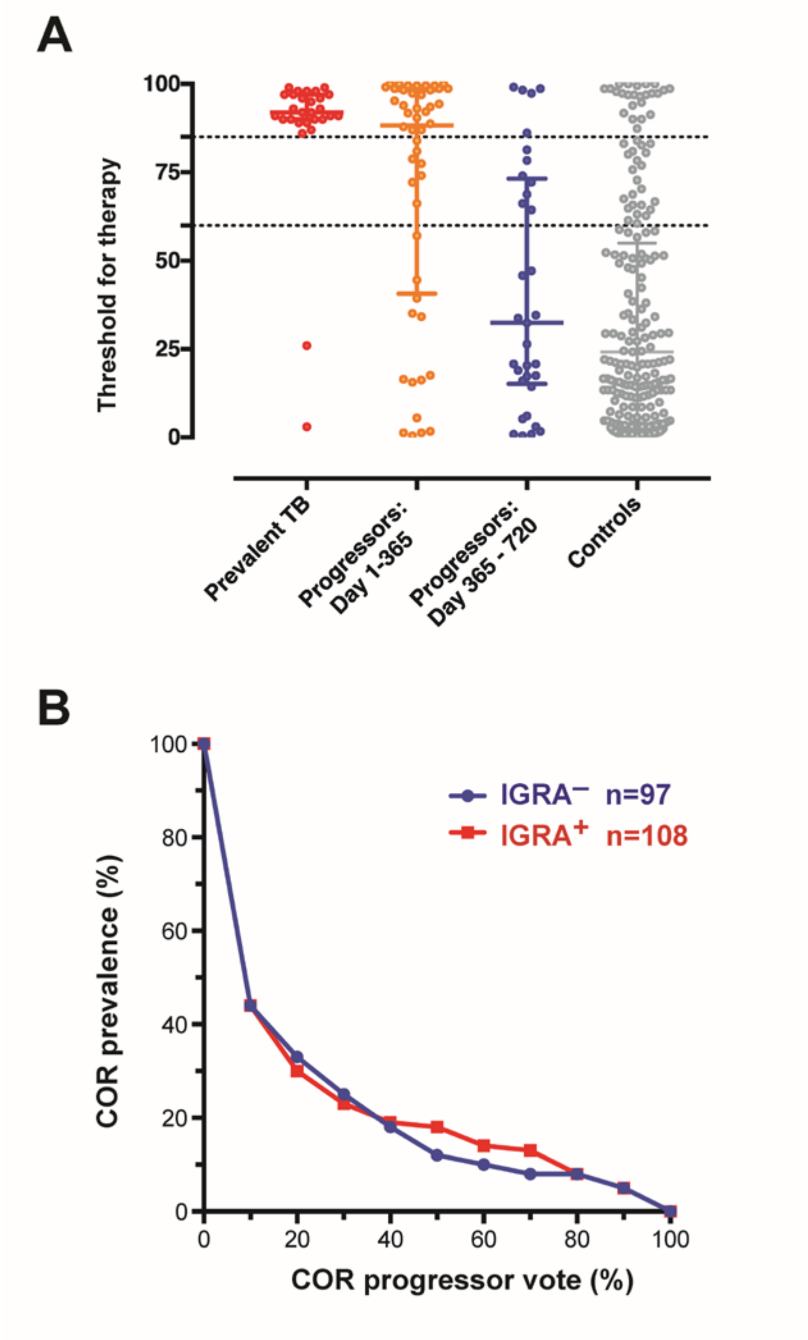

Figure S2. Clinical trial designs. (A) Diagram of a CORTIS trial using a fully randomized design. All participants are randomized to treatment or no treatment, regardless of biomarker status. (B) Diagram of a CORTIS trial using an enrichment design. Only high-risk individuals (i.e. COR$^+$) are enrolled; participants are randomized to treatment or no treatment. (C) Diagram of a CORTIS trial using a biomarker strategy design. The test-and-treat *strategy* is randomized to study participants; participants in one group are provided the standard of care (SOC, i.e. no risk-targeted treatment strategy) while participants in the other are provided treatment based on their biomarker status (i.e. treatment for COR$^+$ only).

A Fully randomized trial design

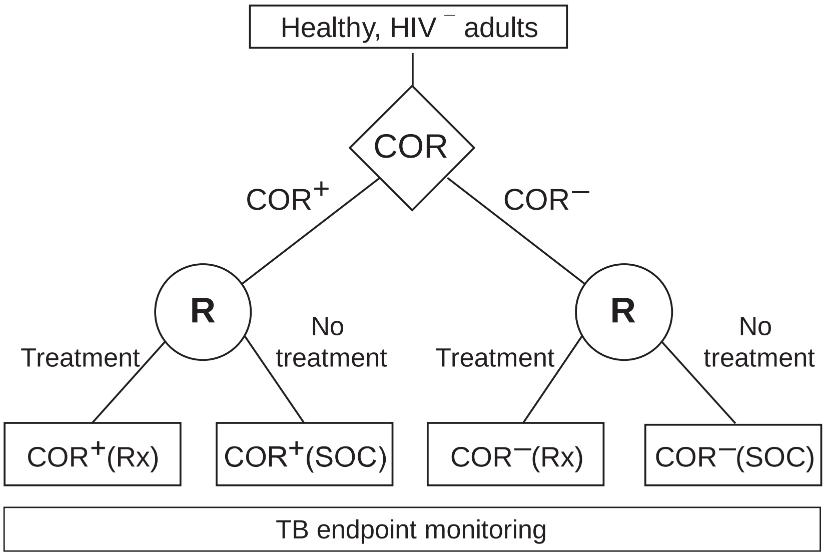

B Enrichment trial design

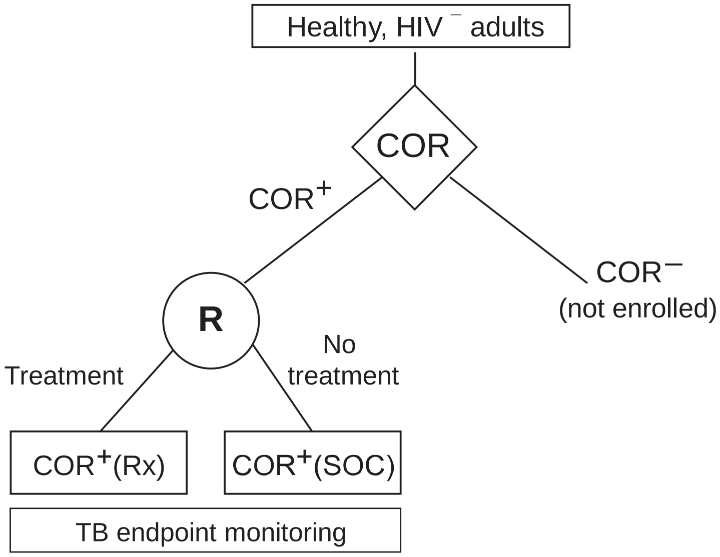

C Biomarker strategy design

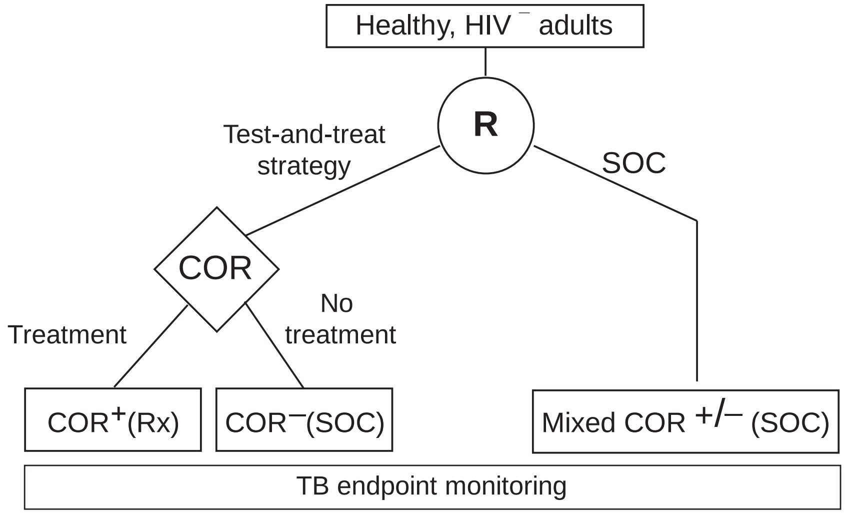

Figure S3. Evaluation of CORTIS objectives in simulated trials. Primary analyses to evaluate treatment efficacy (A), COR relative-risk (B) and strategy efficacy (C) were conducted on 10K stochastically simulated trials. Solid lines show the mean across simulations of the estimate of the parameter over the course of follow-up. Shaded regions indicate the mean of the 95% confidence interval.

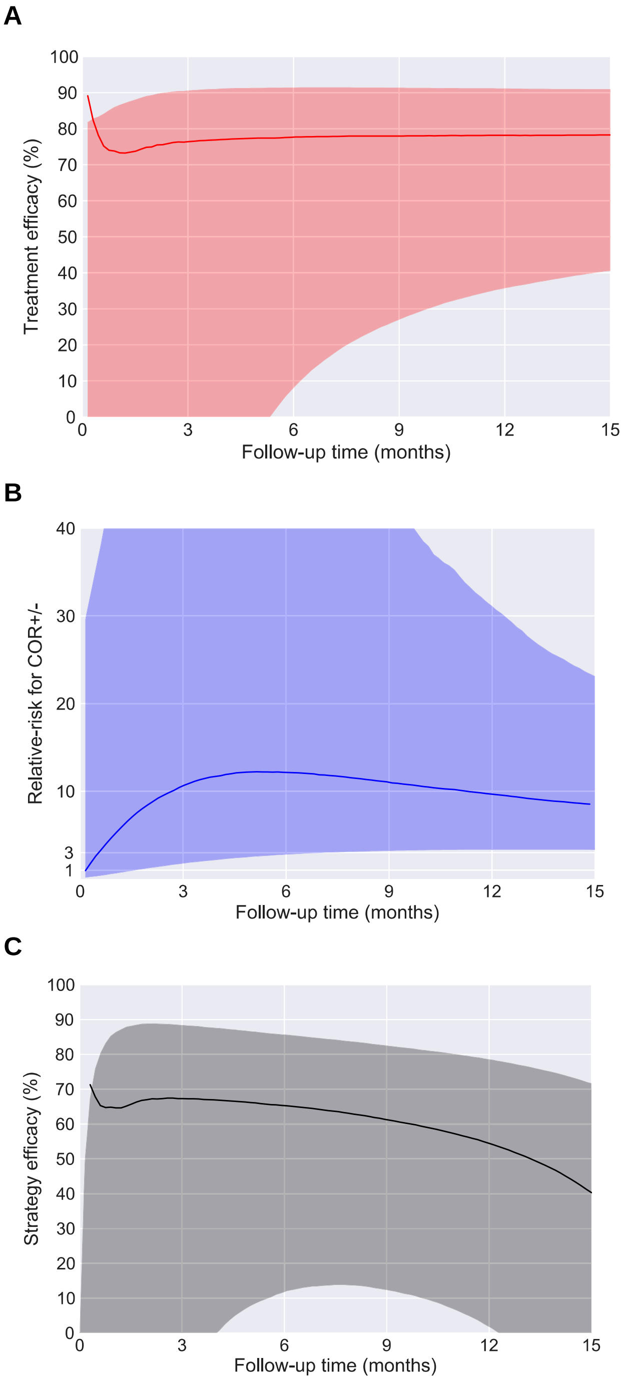

Supplementary Table

Table S1. Simulation Parameters

Parameter description	Value
Total COR+ participants enrolled	1500
Total COR- participants enrolled	1700
Total COR+ participants treated	500
Rate of screening (per week)	210
COR prevalence (%)	15
Duration of follow-up (months)	15
Rate of participants lost to follow-up (%)	10
Treatment efficacy (%)	80
Peak/initial RR_{COR}	15
RR_{COR} decay constant (months)	12
Overall TB incidence (cases per year)	1.1

Supplementary References

1. Mahomed H, Ehrlich R, Hawkridge T, Hatherill M, Geiter L, Kafaar F, et al. TB incidence in an adolescent cohort in South Africa. PLoS One. 2013;8(3):e59652.

2. Aalen O. Nonparametric inference for a family of counting processes. The Annals of Statistics. 1978:701-26.

3. Greenwood M. The Natural Duration of Cancer. Reports of Public Health and Medical Subjects. 1926;33.

4. Borgan O, Liestol K. A Note on Confidence-Intervals and Bands for the Survival Function Based on Transformations. Scandinavian Journal of Statistics. 1990;17(1):35-41.

5. Zak DE, Penn-Nicholson A, Scriba TJ, Thompson E, Suliman S, Amon LM, et al. A blood RNA signature for tuberculosis disease risk: a prospective cohort study. Lancet. 2016;387(10035):2312-22.
